# A new scoring model predicting macroscopic vascular invasion of early-intermediate hepatocellular carcinoma

**DOI:** 10.1097/MD.0000000000013536

**Published:** 2018-12-10

**Authors:** Yao Liu, Le Sun, Fangyuan Gao, Xue Yang, Yuxin Li, Qun Zhang, Bingbing Zhu, Shuaishuai Niu, Yunyi Huang, Ying Hu, Ying Feng, Yuyong Jiang, Xianbo Wang

**Affiliations:** a Center of Integrative Medicine, Beijing Ditan Hospital, Capital Medical University; b Department of Gastroenterology, Dongzhimen Hospital, Beijing University of Chinese Medicine, Beijing, China.

**Keywords:** Barcelona clinic liver cancer staging, hepatocellular carcinoma, macroscopic vascular invasion, predictive scoring model

## Abstract

Macroscopic vascular invasion cannot be properly predicted in advance in hepatocellular carcinoma patients based on clinical characteristics and imaging features.

To develop a predictive scoring model of macroscopic vascular invasion in hepatocellular carcinoma patients after transcatheter arterial chemoembolization combined with radiofrequency ablation based on specific laboratory and tumor indicators.

A predictive scoring model, which estimates the incidence of macroscopic vascular invasion at 1-year follow-up, was constructed based on a derivation cohort of 324 patients with hepatocellular carcinoma; a validation cohort of 120 patients was prospectively included. The prognostic value of the scoring model was determined by concordance index, time-dependent receiver operating characteristics, and calibration curves.

Cox multivariate analysis of the derivation cohort identified prothrombin time, aspartate aminotransferase, and Barcelona clinic liver cancer (BCLC) staging as independent predictive factors of macroscopic vascular invasion. The areas under the receiver operating characteristic curves of the predictive scoring model were 0.832 and 0.785 in the derivation and validation cohorts, respectively, and the calibration curves fitted well. Kaplan–Meier analysis showed that the incidence of macroscopic vascular invasion was significantly higher in the high-risk group (score 0–2) than in the low-risk group (score 3–4) in both the derivation and validation cohorts (*P* < .0001 and *P* = .0008, respectively).

The predictive scoring model enables the accurate prediction of macroscopic vascular invasion incidence 1 year in advance in hepatocellular carcinoma patients who undergo transcatheter arterial chemoembolization combined with radiofrequency ablation.

## 1 Introduction

Hepatocellular carcinoma (HCC) is the 5th most common malignant tumor worldwide.^[1,2]^ Surgical resection and liver transplantation are the main approaches for treating patients with HCC. Unfortunately, resection is only available for approximately 20% patients with HCC.^[3]^ Radiofrequency ablation (RFA) has been proposed as a new therapy owing to its safety and effectiveness for patients with early-intermediate stage HCC.^[4,5]^ Transcatheter arterial chemoembolization (TACE) has also been used to treat unresectable HCC with high accuracy, minimal invasiveness, reduplicated operation, and good tolerance.^[6]^ However, both RFA and TACE have limitations. Compared with the use of TACE or RFA alone, the combination of TACE and RFA for treating HCC is reported to have a relatively high local response rate.^[5]^ Therefore, the combination of TACE and RFA has been widely applied as the main treatment approach for patients with early-intermediate stage HCC. However, approximately 68.9% of patients show tumor recurrence after RFA, which is higher than the rate for liver transplantation and surgical resection.^[7–9]^

Macroscopic vascular invasion (MVI) into the portal vein, hepatic vein, and inferior vena cava appear to be highly correlated with the degree of tumor malignancy,^[10,11]^ which plays an important role in the prognosis of HCC patients. Many studies have shown that MVI is an independent predictor of recurrence and poor outcomes in patients with HCC.^[12,13]^ Portal vein tumor thrombosis (PVTT) is observed in 10% to 60% of HCC patients and can lead to liver dysfunction, portal hypertension, ascites formation, variceal rupture, hepatic encephalopathy, and/or death.^[14–17]^ According to the American Association for the Study of Liver Diseases and the Barcelona clinic liver cancer (BCLC) staging system and treatment strategy, HCC that is associated with MVI is considered an advanced stage that requires aggressive treatment.^[18]^ Sorafenib, a small tyrosine kinase inhibitor molecule, has been used as a palliative treatment, and the median survival time of patients with MVI who are treated with this drug is only 8.1 months.^[16,19]^ Therefore, it is essential to accurately predict the occurrence of MVI before treatment.

Currently, MVI can only be diagnosed by medical imaging examination, such as ultrasound, computed tomography (CT), magnetic resonance imaging (MRI), and angiography. Previous studies have shown that tumor size, Edmondson–Steiner histological grade, number of nodules, and alpha-fetoprotein (AFP) level are associated with PVTT.^[20]^ While low albumin levels, tumor size >5 cm, metastases, ascites, AFP >1000 ng/mL, and hypersplenism have been found to be independent predictive factors of MVI,^[21–23]^ these indicators cannot properly predict the risk of MVI. In our study, we retrospectively investigated clinical characteristics and imaging features of HCC patients and established a predictive scoring model through measurement of independent predictors of MVI. Our predictive scoring model allows more accurate individualized risk estimates for the incidence of MVI in HCC patients after TACE combined with RFA.

## 2 Methods

### 2.1 Patient selection

Patients with an initial diagnosis of HCC in BCLC A or B stages who underwent TACE combined with RFA were included in our study. Indications for using TACE combined with RFA for HCC patients were presence of Child–Pugh A or B liver function. A total of 324 patients were enrolled in the derivation cohort at the Beijing Ditan Hospital (Beijing), Capital Medical University between June 2008 and May 2014, and 120 patients were prospectively enrolled in the validation cohort between June 2014 and May 2016. The diagnosis of HCC relied on the recommendations of the American Association for the Study of Liver Diseases.^[24]^ The diagnosis of MVI was made using contrast-enhanced imaging (CT scan or MRI) when a filling defect was shown in the portal vein, hepatic vein, or inferior vena cava, and the emboli was enhanced in the same or similar way as the primary liver cancer.^[25]^ Patients with autoimmune liver disease, hepatitis A, D, or E, syphilis, acquired immune deficiency syndrome, or other primary malignancies, were excluded from our study. Patients were also excluded if they had incomplete data or lacked follow-up. Patient data was anonymized before analysis. This project was approved by the ethics committee of the Beijing Ditan Hospital, Capital Medical University. As this study had a retrospective design, we could not obtain informed consent from all patients. However, to protect patient privacy, we anonymized and deidentified all patient records and information before the analysis.

Indications for using TACE combined with RFA for HCC patients were as follows: presence of Child–Pugh A or B liver function, a total bilirubin level of 42 μmol/L, and normal serum creatinine level. A CT scan was conducted one week later to estimate the treatment effect of TACE. Patients with active local tumor lesions underwent RFA therapy.

### 2.2 Data collection

Clinical data included demographic status (age, sex, and family history of HCC), etiology (HBV-Ag, anti-HCV, and alcohol consumption), liver function (alanine aminotransferase [ALT], aspartate aminotransferase [AST], ɣ-glutamyl transpeptidase [GGT], total bilirubin [TBIL], serum albumin [ALB], prothrombin time [PT], Child–Pugh class), routine blood tests (white blood cell [WBC] count), complications (ascites, hypersplenism), and tumor-related indicators (AFP, tumor number, largest tumor diameter, and BCLC staging). The data were collected at the time of HCC diagnosis.

### 2.3 Follow-up

Clinical evaluation included liver function, AFP, and contrast-enhanced CT or MRI scan every 2 to 3 months for 1 year, starting from the baseline. In terms of local lesion recurrence, intrahepatic distant recurrence and extrahepatic recurrence were further developed subsequently. Thus, the corresponding treatment methods, such as RFA, TACE, and conservative treatments, were proposed according to the features of recurrent tumors and the patient's liver function condition based on the individual's requirements.

### 2.4 Determination of cut-off value

Using 1-year incidence of MVI as an endpoint, grading of ALT, AST, GGT, TBIL, ALB, WBC count, and PT were determined by receiver operating curve (ROC) curve analyses. The results showed optimal cut-off values of 70 IU/L, 93 IU/L, 87 IU/L, 23 μmol/L, 34 g/L, 2.6 × 10^9^/L, and 13.4 seconds, respectively, corresponding to the maximum Youden index value (Table 1).

**Table 1:**
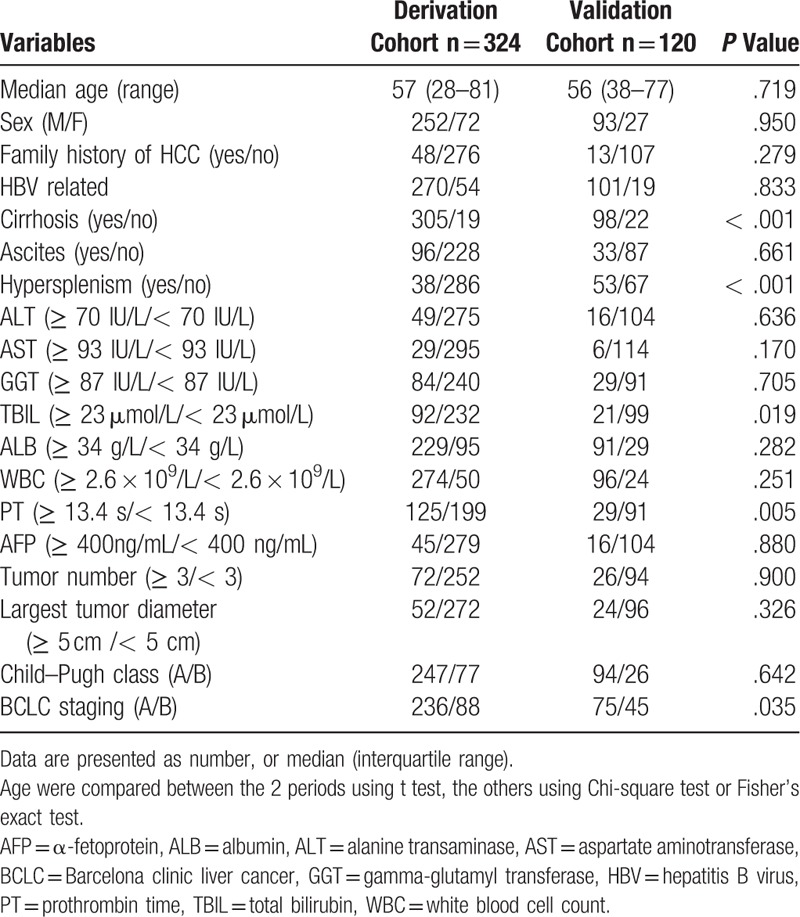
Clinical characteristics of the HCC patients.

### 2.5 Statistical analysis

Demographic and clinical characteristics were summarized as the median and range, number. Most experimental data had been converted to categorical variables based on cut-off values, which were calculated based on maximum Youden index (Sensitivity + Specificity -1) values. Categorical variables were compared between the 2 periods using the Pearson chi-squared test (all of the theoretical number ≥5 and the total sample size ≥40). Continuous variable age was compared between samples using the Student *t* test, as data were normally distributed. The occurrence time of MVI was calculated from the date of diagnosis of HCC to MVI positive or the last day of follow-up, according to the Kaplan–Meier method, and compared using log-rank test. Univariate and multivariate Cox proportional hazards regression analyses (stepwise forward method) were performed to identify significant factors predicting the risk of MVI, at a *P* < .05 (2-tailed). The predictive scoring model was established by each predictor according to the β coefficient in the multivariate Cox regression analyses. The predictive accuracy of the scoring model was measured using the concordance index (c-index). The time-dependent receiver operating characteristics curve (tdROC) evaluated the accuracy of quantitative markers for time-varying outcomes. The area under time dependent ROC curve (tdAUC) and the calibration curves were also estimated to assess the performance of the scoring model. MVI incidence curves were constructed by the Kaplan–Meier method and compared by log-rank test. Analyses were performed using SPSS 22.0 statistical package (IBM, Armonk, NY) and RMS packages in R version 3.0.2.

## 3 Results

### 3.1 Characteristics of the derivation and validation cohorts

A total of 444 patients diagnosed with HCC without MVI after TACE combined with RFA were included in the study. In the derivation cohort, most individuals were male, with median age of 57 years (range: 28–81 years) and were positive for HBV surface antigen (83.3%). The clinical characteristics of the derivation and validation cohorts are summarized in Table 1. Most subjects had < 3 tumors, tumors <5 cm in diameter, AFP < 400 ng/mL, Child–Pugh class A, and no ascites. The number of patients with optimal cut-off values for ALT, AST, GGT, TBIL, ALB, WBC, and PT are presented in Table 1.

### 3.2 Prognostic factors for MVI

Results of univariate Cox regression analyses showed that ascites, ALT, AST, TBIL, WBC, PT, and BCLC obtained from the derivation cohort were predictive factors of MVI (Table 2). These factors were included in the multivariate Cox regression analyses. Furthermore, PT, AST, and BCLC B stage were selected as significant prognostic factors of MVI by forward selection. The cut-off values of PT and AST were 13.4 seconds and ≥93 IU/L, respectively, according to ROC analysis (Table 3).

**Table 2:**
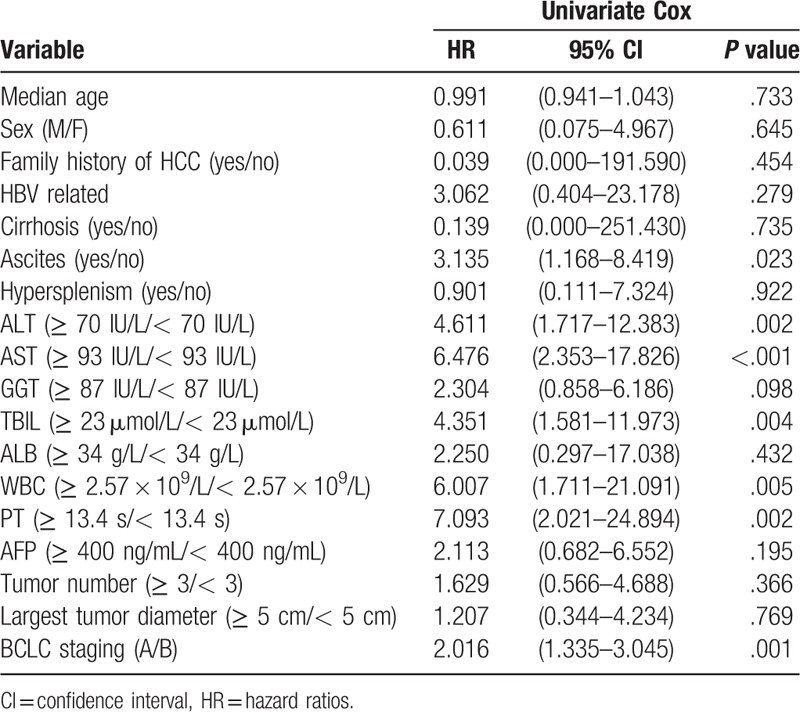
Predictors for MVI of patients with HCC on univariate cox regression analysis in the derivation cohort.

**Table 3:**
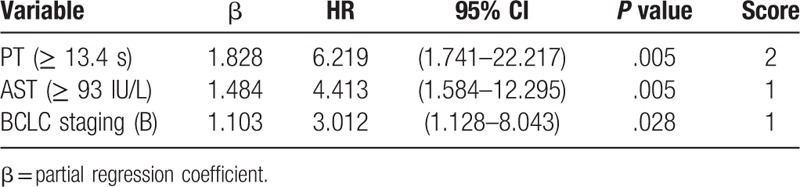
Predictors for MVI of derivation cohort on multivariate cox regression analysis.

### 3.3 Establishment and validation of a predictive scoring model for MVI

**Figure 1:**
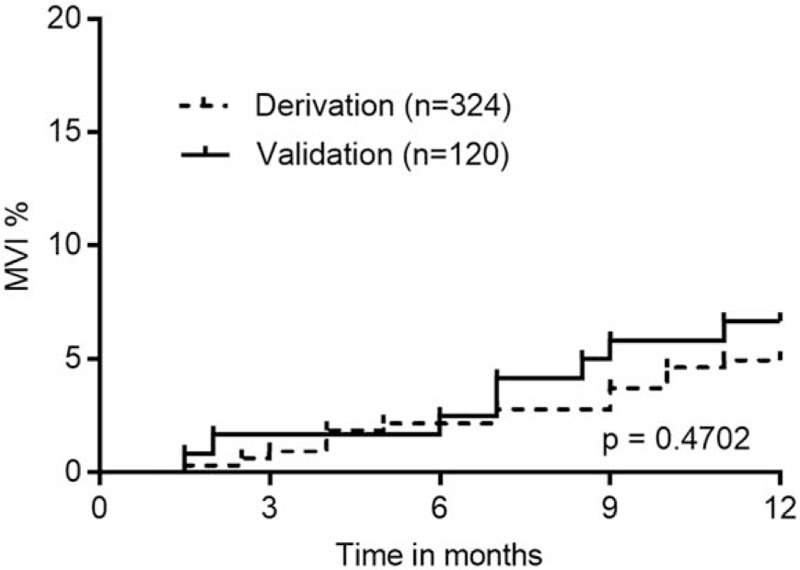
Kaplan–Meier analysis for the incidence of macroscopic vascular invasion (MVI). The incidence of MVI in the derivation cohort at 1-year follow-up was similar to that in the validation cohort (*P* = .4702). MVI = macroscopic vascular invasion.

**Figure 2:**
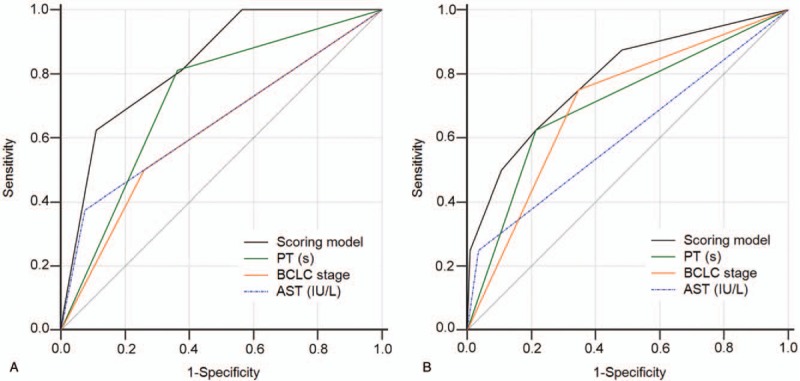
Receiver operating characteristic (ROC) curves of the predictive scoring model for the (A) derivation cohort and (B) validation cohort. ROC = receiver operating characteristic.

Kaplan–Meier analysis showed that the MVI incidence in patients who received combination therapy of TACE with RFA were similar in the derivation and validation cohorts after one year (*P* = .4702) (Fig. 1). We established a predictive scoring model for MVI according to the β value of each independent predictive factor in the derivation cohort (the β coefficient of each predictor divided by 1.103). Points were assigned to each predictive factor as follows (Table 3): PT ≥13.4 seconds (β = 1.828, score = 2), AST ≥93 IU/L (β = 1.484, score = 1), and BCLC B stage (β=1.103, score = 1). The total predictive score of each HCC patient was defined as the sum of scores for each prediction factor, which ranged from 0 to 4 points. Based on ROC curve analysis, the AUROC was 0.832 (95% CI, 0.787–0.871) in the derivation cohort (Fig. 2). In the validation cohort, the AUROC for the predictive scoring model was 0.785 (95% CI, 0.700–0.854) (Fig. 2).

**Figure 3:**
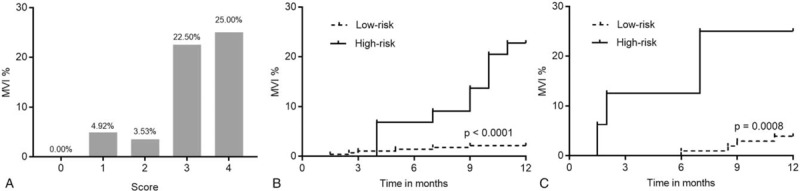
The bars show the proportion of macroscopic vascular invasion (MVI) at 1-year follow-up for each score category in the derivation cohort (**A**). The incidence of MVI in the high-risk group (score 3–4) was significantly higher than that in the low-risk group (score 0–2) over the 12-month follow-up period in the derivation (**B**) and the validation (**C**) cohorts (*P* < .0001 and *P* = .0008, respectively). MVI = macroscopic vascular invasion.

To further assess the relationship between the scoring model and MVI rate, all patients were analyzed. Based on observations, MVI rates increased as scores increased, from 0.00%, when the score value was 0, to 4.92%, 3.53%, 22.50%, and 25.00%, when score values were 1, 2, 3, and 4, respectively, in the derivation cohort (Fig. 3A). Kaplan–Meier analysis showed that the MVI incidence in patients of the high-risk group (score 3–4), was significantly higher at 12 months after diagnosis of HCC than that found in patients with a score of 0–2 in both the derivation and validation cohorts (*P* < .0001 and *P* = .0008, respectively, Fig. 3B, C).

### 3.4 Predictive value of the new scoring model

**Figure 4:**
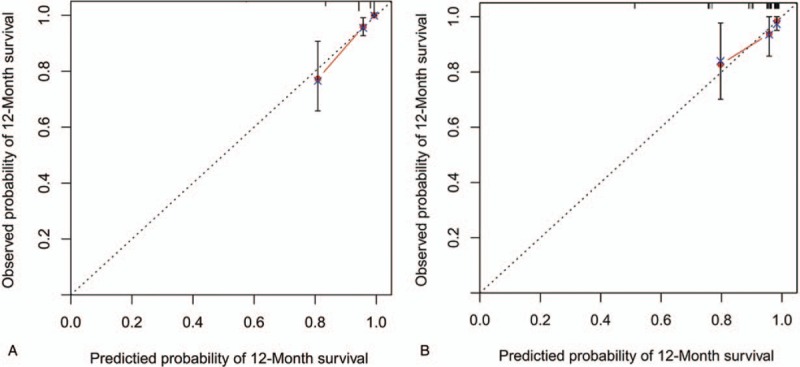
Calibration plot of the scoring model for macroscopic vascular invasion (MVI) rates in the derivation cohort (**A**) and validation cohort (**B**), in which the predicted probability of survival was compared with actual survival, MVI = macroscopic vascular invasion.

The new scoring model had a c-index of 0.820 (95% CI, 0.737–0.903) in the derivation cohort, and 0.786 (95% CI, 0.625–0.947) in the validation cohort. The calibration curves showed that the predicted MVI probabilities were in agreement with the actual results observed in both the derivation (Fig. 4A) and validation cohorts (Fig. 4B).

**Figure 5:**
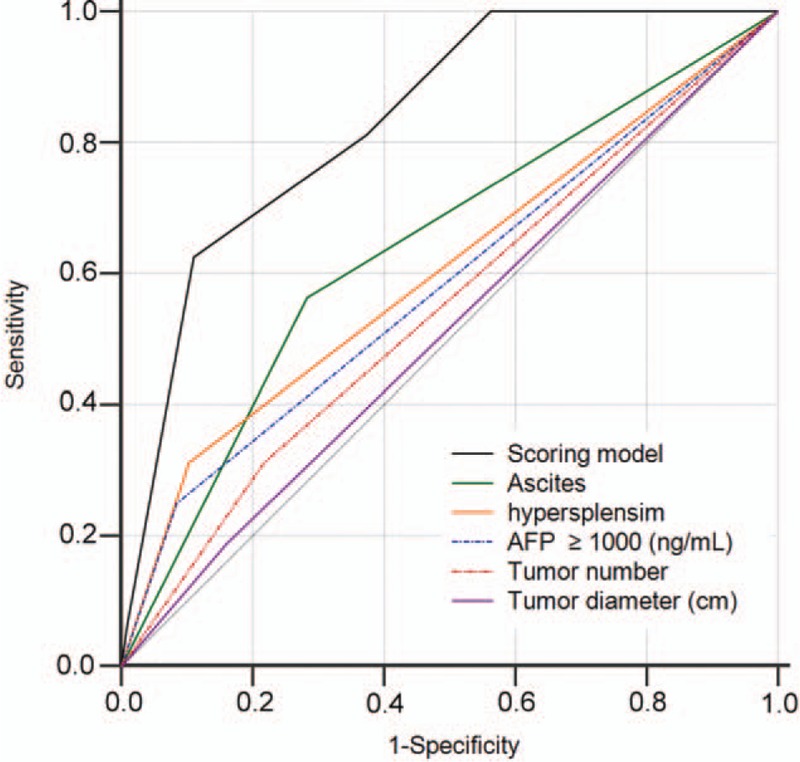
ROC analysis of MVI incidence at 12 months in the derivation cohort. The area under the curve (AUC) of the scoring model developed in the study was greater than that of previously reported indicators. AUC = area under the curve, MVI = macroscopic vascular invasion, ROC = receiver operating characteristic.

Using tdROC curves, the predictive value of the new scoring model for the incidence of MVI was compared to the predictive value based on tumor number ≥3, tumor size ≥5 cm, AFP ≥1000 ng/mL, hypersplenism, and ascites. As shown in Figure 5, we found that the established model had the highest tdAUC (0.832) for serum biochemical markers and tumor characteristics, compared with that for previously reported indicators, including tumor number ≥3 (0.547, *P* = .0005), tumor diameter ≥5 cm (0.514, *P* < .0001), AFP ≥1000 ng/mL (0.583, *P* = .0020), hypersplenism (0.604, *P* = .0026), and ascites (0.640, *P* = .0031).^[17–20]^

### 3.5 Comparison of the new scoring model with BCLC staging

**Figure 6:**
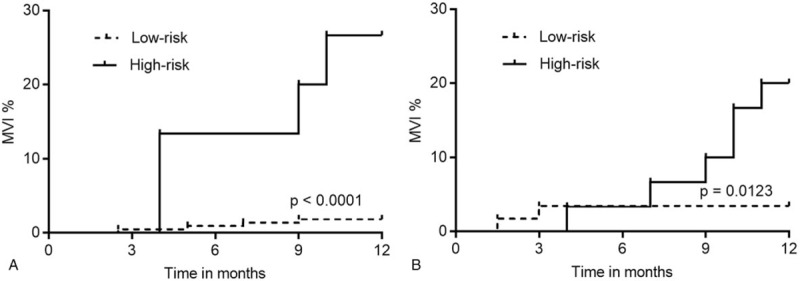
The MVI incidences of BCLC A and B stage. The incidence of MVI in the high-risk group was significantly higher than that in the low-risk group over the 12-month follow-up period for patients with BCLC A stage (A, *P* < .0001) and B stage (B, *P* = .0123). BCLC = Barcelona clinic liver cancer, MVI = macroscopic vascular invasion.

In the derivation cohort, Kaplan–Meier analysis showed that the MVI incidence for patients in the high-risk group (scores of 3–4) was significantly higher at 12 months after diagnosis of HCC than that for patients with a score of 0–2 in the BCLC A and B stages (*P* < .0001 and *P* = .0123, respectively, Fig. 6A, B). Univariate and multivariate Cox regression analyses showed that BCLC staging was a significant prognostic factor of MVI; tdAUC was 0.620 in the derivation cohort and 0.701 in the validation cohort.

To compare the predictive effect of the new scoring model with BCLC staging for MVI, we calculated the additive net reclassification index (NRI) and absolute NRI, which are the most widely used summary statistics to summarize the extent of reclassification^[26]^ (Table 4). An additive NRI of 27 suggested improved prediction when prothrombin time and aspartate aminotransferase were added to BCLC staging. An absolute NRI was 48 of 324, or approximately 15%. The overall reclassification after adding prothrombin time and aspartate aminotransferase to BCLC staging improved the risk prediction in 15% of the patients, compared to the use of BCLC staging alone.

**Table 4:**
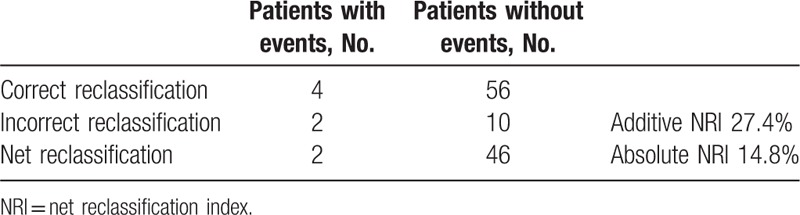
Additive NRI and absolute NRI analysis.

## 4 Discussion

The presence of MVI suggests poor prognosis for HCC patients and has adverse effects on HCC recurrence. The formation of PVTT is related to the blood supply, physiological function, and anatomical position of portal vein. The liver function of HCC patients is worse, the chance of portal hypertension is higher, the tolerance to treatment is lower, and the prognosis is poor when HCC is followed by PVTT.^[27]^ Therefore, it is necessary to achieve accurate prediction of MVI incidence in patients with HCC, according to clinical features.

In the present research, clinical and radiologic characteristics of HCC patients in BCLC stages A or B after TACE combined with RFA were incorporated into the multivariate Cox regression analyses. We found that PT, AST, and BCLC staging were independent predictors of MVI, and that these predictors were observed 1 year before MVI diagnosis. Based on these 3 parameters, we established a scoring model for predicting MVI risk. For clinical applications of the model, we summarized the cut-off value. Patients with a score of 3–4 (44 of 324, 13.6%), and 0–2 (280 of 324, 86.4%) were classified as high-risk and low-risk subgroups, respectively. Furthermore, the predictive value of the scoring model was found to be superior to that of previously reported indicators, including tumor characteristics, hypersplenism, and ascites. The new scoring model also improved the risk prediction for 15% of patients, compared with BCLC staging, which was an independent risk factor for MVI in this study.

Considering the MVI incidence, the negative and positive predictive values were 93.8% and 47.2%, respectively, in the derivation cohort, which means that the percentage of patients of the low-risk group who will not develop MVI in 1 year accounts for 93.8%. Likewise, the positive predictive value of 47.2% means that, among patients of the high-risk group, 47.2% will develop MVI in 1 year, which is acceptable. Therefore, it is of great importance that nearly half of the patients (47.2%) in the high-risk group receive a more suitable treatment. Liver transplantation (LT) and RFA are not recommended as initial treatments if there is a high risk of MVI occurrence in patients with HCC because of the high HCC recurrence rate after such therapies.^[28,29]^ For patients with intermediate-stage (BCLC B) HCC, the only recommended treatment strategy is TACE.^[30]^ However, Kamiyama et al evaluated 297 patients with BCLC B HCC who underwent curative hepatectomy and observed a recurrence of 71.0% and a 5-year overall survival (OS) rate of 54.3%, which are acceptable.^[31]^ Moreover, studies^[32–34]^ have shown that liver resection can improve OS and reduce local recurrence rate better than RFA in early-stage HCCs. Liu et al showed that surgical resection resulted in better OS compared with sequential treatment with TACE and RFA for HCC within the Milan criteria.^[35]^ Therefore, surgical resection may be recommended as a surrogate for TACE combined with RFA, to prevent recurrence in patients with HCC and high risk of MVI.^[36]^ Based on the 3 predictors of our study, the scoring model might serve as a tool to assess patients with different risks of MVI 1 year in advance after TACE combined with RFA, which may help guide treatment decisions.

Aspartate aminotransferase (AST) is an enzyme found mainly in the liver. Additional AST is released into the bloodstream when the liver is diseased or damaged, causing levels of the enzyme to increase. Therefore, blood AST levels are directly related to the extent of liver damage.^[37]^ Moreover, studies have shown that serum AST changes are closely correlated with HCC growth.^[38,39]^ Our study showed that a high AST level is a predictive risk factor for MVI.

Moreover, our study showed that a high prothrombin time (PT) is an independent predictive risk factor associated with MVI in HCC patients. PT is an important indicator of hepatic coagulation and reserve function. Hemostatic disturbance is more complex in HCC patients with liver cirrhosis, especially those with impaired liver function.^[40,41]^ A retrospective study observed that PT is an independent risk factor for HCC prognosis: patients with increased PT had significantly shorter OS times.^[42]^ The exact relationship between PT and MVI is still not clearly understood.

Our study has the following limitations: First, the scoring model was established based on data from a single center, and validation of the scoring model at other institutions will be necessary. However, our validation cohort was prospectively investigated, which allows a proper assessment of the scoring model accuracy. Second, the limited follow-up time and the low incidence of MVI in the derivation cohort, which was about 5% in 1 year, might have influenced the results of MVI predictors. However, the establishment of a predictive MVI scoring system may not be significant, since as follow-up duration increase, mortality rates also increase, which about to reach 73% in the 5 years based on our study data. In addition, the median survival time among HCC with MVI patients was only 2–4 months. Therefore, the short-term MVI risk evaluation was indeed valuable. Despite these limitations, we have established the 1st scoring model for predicting MVI in HCC patients after TACE combined with RFA, which may help guide treatment decisions.

## Author contributions

**Conceptualization:** Ying Feng, Yuyong Jiang, Xianbo Wang.

**Data curation:** Yao Liu, Le Sun, Fangyuan Gao, Bingbing Zhu, Shuaishuai Niu, Yunyi Huang, Yuyong Jiang, Xianbo Wang.

**Formal analysis:** Yao Liu, Fangyuan Gao, Qun Zhang.

**Investigation:** Ying Feng.

**Project administration:** Yuxin Li.

**Resources:** Yuxin Li.

**Software:** Xue Yang.

**Supervision:** Ying Hu.

**Writing – original draft:** Yao Liu.

**Writing – review & editing:** Yao Liu.
